# Hypometabolic patterns of focal cortical dysplasia in PET-MRI co-registration imaging: a retrospective evaluation in a series of 83 patients

**DOI:** 10.3389/fnins.2023.1173534

**Published:** 2023-09-25

**Authors:** Xiu Wang, Wenhan Hu, Xiaoqiu Shao, Zhong Zheng, Lin Ai, Lin Sang, Chao Zhang, Jian-guo Zhang, Kai Zhang

**Affiliations:** ^1^Department of Neurosurgery, Beijing Tian Tan Hospital, Capital Medical University, Beijing, China; ^2^Stereotactic and Functional Neurosurgery Laboratory, Beijing Neurosurgical Institute, Capital Medical University, Beijing, China; ^3^Beijing Key Laboratory of Neurostimulation, Beijing, China; ^4^Department of Neurology, Beijing Tian Tan Hospital, Capital Medical University, Beijing, China; ^5^Epilepsy Center, Medical Alliance of Beijing Tian Tan Hospital, Peking University First Hospital Fengtai Hospital, Beijing, China; ^6^Department of Nuclear Medicine, Beijing Tian Tan Hospital, Capital Medical University, Beijing, China

**Keywords:** PET-MRI co-registration, focal cortical dysplasia, epilepsy, hypometabolic pattern, PET

## Abstract

**Objective:**

To characterize the PET-MRI co-registration of hypometabolic patterns in focal cortical dysplasia (FCD) types I and II and provide some suggestions in presurgical evaluation of epilepsy surgery.

**Methods:**

We retrospectively analyzed PET-MRI co-registration imaging data from a cohort of 83 epilepsy patients with histologically confirmed FCD types I and II. Hypometabolic patterns were classified into 4 types: bottom of sulcus hypometabolism (BOSH), single island of sulcus hypometabolism (SIOS), single gyrus or sulcus hypometabolism (SGOS), and multiple gyri and sulci hypometabolism (MGOS).

**Results:**

Most of cases that were overlooked by conventional MRI and PET evaluation but positive in PET-MRI co-registration were focalized lesions in dorsolateral frontal lobe (9/15) and FCD type IIa was the most prevalent pathological type (11/15). The FCD histological types (*p* = 0.027) and locations (*p* < 0.001) were independent predictors of PET-MRI co-registration hypometabolic patterns. Focalized hypometabolic patterns (BOSH, SIOS, SGOS) were primarily observed in the frontal lobe (33/39) and FCD type II (43/62) and extensive pattern (MGOS) in temporal lobe (18/20) and FCD type I (16/21; *p* < 0.005).

**Conclusion:**

PET-MRI co-registration enhanced the detection of FCD type IIa compared with conventional MRI and PET reading. The hypometabolic patterns of FCD type I and temporal lobe FCD were more extensive than those of FCD type II and frontal lobe FCD, respectively. The predilection of focalized hypometabolic patterns in frontal lobe FCD suggested that subtle lesions should be checked carefully in patients with suspected frontal lobe epilepsy.

## Introduction

Focal cortical dysplasia (FCD) represents one of the most frequent pathologies in epilepsy surgery (Chassoux et al., 2010). Patients with MRI positive FCD were reported to have better seizure outcome than those with MRI negative FCD (Fauser et al., 2015). Recent findings showed an interictal [18F] fluorodeoxyglucose-positron emission (^18^FDG-PET) was reliable in localizing FCD, including MRI negative FCD (Chassoux et al., 2010). The complete removal of the dysplastic cortex was the main predictor of a favorable surgical outcome (Kim et al., 2009; Lerner et al., 2009). Accordingly, the co-registration of PET and structural MRI images could not only improve the sensitivity of FCD detection but also clearly and precisely delineate the FCD in the preoperative evaluation prior to epilepsy surgery. According to the hypometabolic area of PET-MRI co-registration, the epileptogenic lesions were strictly co-localized with the focalized hypometabolic areas, especially in FCD type II (Chassoux et al., 2010; Sukprakun and Tepmongkol, 2022); while in temporal lobe FCD, the hypometabolic areas were usually larger than the actual lesions (Yokota et al., 2020). Thus we proposed that the extension of the hypometabolic area might be correlated with the FCD subtypes and other anatomical factors.

For the quantitative PET analysis, limited detection ability was demonstrated for small FCD lesions (Hu et al., 2018; Tomás et al., 2019). PET-MRI co-registration was observed to be superior in FCD detection than the statistical parametric mapping (SPM) analysis of PET (SPM-PET). In the present study, we trying to summarize the hypometabolic patterns of FCD according to the PET-MRI co-registration visual analysis. The hypometabolic patterns were determined according to the extension of the hypometabolism. The results will demonstrate the influencing factors for the hypometabolic extension and guide the clinicians for PET-MRI co-registration interpretation.

## Materials and methods

### Patient selection

Among all patients who underwent a resective epilepsy surgery in our department between January 2015 and August 2017, we analyzed 83 consecutive patients with pathologically proven FCD types I and II. We excluded patients with hemimegalencephaly, serious systemic diseases, drug or alcohol abuse, brain injury or cerebrovascular disease within the previous 2 years. FCD type III were not included because temporal neocortical hypometabolism might be partly influenced by epileptic discharges from abnormal hippocampus. An expert neuropathologist reviewed the pathological diagnoses according to the International League Against Epilepsy (ILAE; Blümcke et al., 2011): FCD type I refers to isolated lesions with radial or tangential dyslamination of the neocortex and FCD type II is characterized by cortical dyslamination and dysmorphic neurons without (Type IIa) or with balloon cells (Type IIb). A detailed pre-surgical evaluation, including medical history, neurological examination, and high-resolution MRI and ^18^FDG-PET, was performed for all patients. This study has been approved by Institutional Review Boards of Beijing Tian Tan Hospital and informed consent was obtained from all individual participants included in the study.

### Imaging data acquisition

The MRI scans were performed on a 3.0-T Siemens Verio scanner (Siemens Medical system, South Iselin, NJ) with a 3D T1 sagittal Magnetization Prepared Rapid Gradient Echo sequence (MPRAGE; TR/TE 1900/2.53, TI 900, matrix 256 × 256, 1.0 mm thickness), a T2 axial (TR/TE 7030/110, matrix 256 × 320, 3 mm thickness), a FLAIR axial sequence (TR/TE 8000/94, TI 2371.5, matrix 424 × 512, 3 mm thickness), a FLAIR sagittal sequence (TR/TE 8000/96, TI 2371.2, matrix 236 × 256, 3 mm thickness), and a FLAIR coronal sequence (TR/TE 8000/96, TI 2371.2, matrix 408 × 512, 3 mm thickness). All MRI scans were reviewed by a trained radiologist, who was blind to the PET results and the localization of the lesion. The MRI imaging features of FCD were classified as negative (normal, non-specialized or ambiguous findings) or positive.

The PET scans were obtained in the interictal state using ^18^F-FDG under standard resting conditions. The ^18^FDG-PET examination was performed using the GE Discovery STPET-CT system (300 mm FOV, matrix 192 × 192, 3.27 mm slice thickness). The ^18^FDG was injected IV at a mean dose of 310 MBq/70 kg body weight. The reconstructed images were corrected for attenuation using transmission scans obtained from a germanium source. No patients had an ictal event less than 6 h before and during the PET scan. The visual PET analysis was performed by a trained examiner, who was blind to the patients and MRI images, using a colored scale that allowed for detection of metabolic changes, and each color corresponded to a 15% variation in the FDG uptake. Areas with asymmetric hypometabolism were considered PET positive, and areas with completely normal and symmetric distribution of cortical metabolism were classified PET negative.

### PET-MRI co-registration and reading

The PET image was co-registered with the MRI structural image in SPM 8 (Institute of Neurology, University College of London, London, United Kingdom), which is implemented in MATLAB 2012 (The MathWorks Inc., Natick, MA, United States). The PET and MRI images were overlaid using the MRIcron software (Chris Roeden, version 11, November 2011). The fused images were displayed as spectrum images, which was similar to the PET alone images. The fusion images were interpreted visually with a common transparency of 60%~80% of the PET image over the MRI image, which could be modified freely from 0% to 100% if required or considered useful by the examiner.

**Figure 1:**
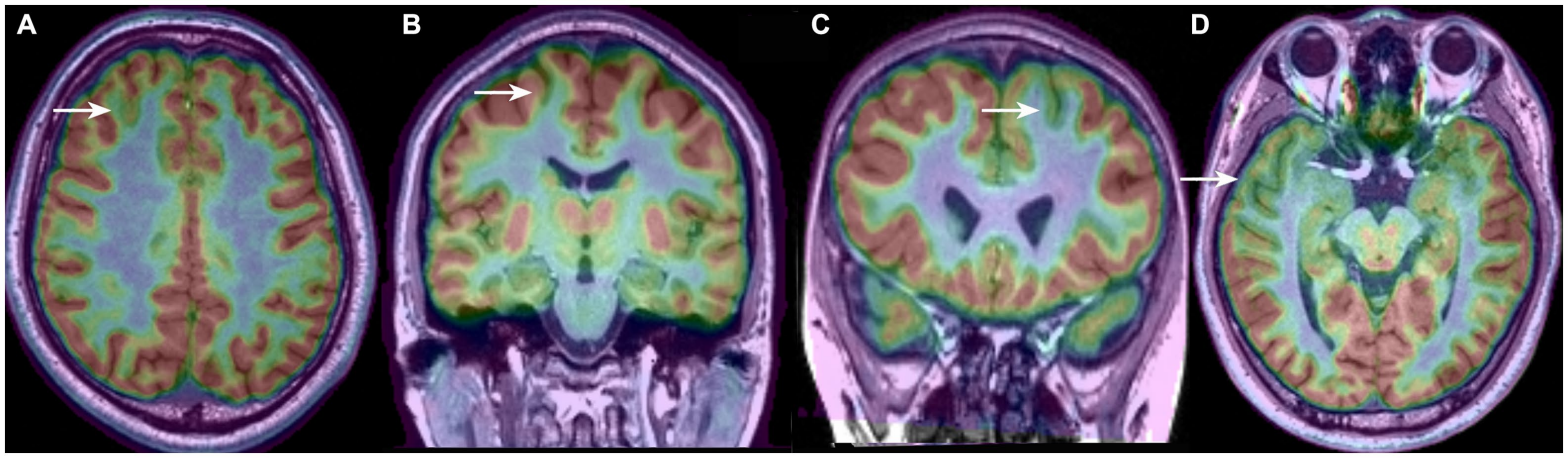
Four types of PET-MRI hypometabolic patterns of FCD: **(A)** bottom of sulcus hypometabolism (BOSH); **(B)** single island of sulcus hypometabolism (SIOS); **(C)** single gyrus or sulcus hypometabolism (SGOS); **(D)** and multiple gyri and sulci hypometabolism (MGOS).

The borders and patterns of the PET hypometabolism area were determined based on the asymmetry of the uptake between the two cerebral hemispheres. To clearly distinguish the area size and distribution feature of the hypometabolic patterns, we classified the PET-MRI co-registration hypometabolic patterns into the following 4 types according to our clinical observation and description in previous literature (Figure 1): bottom of sulcus hypometabolism (BOSH), single island of sulcus hypometabolism (SIOS), single gyrus or sulcus hypometabolism (SGOS), and multiple gyri and sulci hypometabolism (MGOS). BOSH was defined as a hypometabolic lesion that did not extend to the superficial cortex at the surface of the brain. The coronal, sagittal and horizontal images and 3-dimension cerebral surface were carefully evaluated to finally define the individual hypometabolic pattern in PET-MRI co-registration. The PET, MRI and PET-MRI co-registration images were sequentially evaluated by X.W., X.Q.S (epileptologist) and L. A. (neuroimage specialist) independently. Disagreements were resolved by reaching a consensus through discussion, which involved a senior epilepsy neurosurgeon (K. Z). Negativity and positivity referred to the FCD focus resected by surgery regardless of any other nonadjacent metabolic abnormalities. PET hypometabolism was defined as greater than 15% asymmetry and the ambiguous appearance in MRI imaging was considered as MRI negativity.

### Statistical analysis

First, the univariate statistical analysis was applied to analyze the patients’ features according to the hypometabolic patterns of PET-MRI imaging. The Kruskal-Wallis rank sum test or Fisher’s two-tailed exact test was performed depending on the variable types. Then, relevant predictors, according to the univariate analysis, were chosen for multivariable analysis. Multiple logistic regression analyses were used to analyze the factors with a value of *p* ≤ 0.1 in the previous univariate analysis (Lagarde et al., 2016). We fitted the logistic regression analysis to determine the predictors of the PET-MRI hypometabolic patterns. An adjusted (Bonferroni correction) value of *p* < 0.05 was considered statistically significant. The statistical tests were performed with SPSS 24.0 (IBM Corp., Armonk, NY).

## Results

### Patient features

Eighty-three patients were included in this study. Four patients with normal PET-MRI imaging were excluded, and no patients were found to be hypermetabolic in the PET-MRI imaging. FCD diagnosis were the following: FCD type I (21), FCD type II (62; FCD IIa = 33; FCD IIb = 29); the FCD distributions are the following: frontal lobe (39), temporal lobe (20), parietal lobe (14), occipital lobe (6), and insular lobe (4).

The mean seizure onset age was 8.3 ± 6.7 years; the mean surgery age was 17.9 ± 9.4 years and the mean epilepsy duration was 9.6 ± 7.1 years. Four patients had undergone a previous epilepsy surgery, including temporal lobectomy (two cases), frontal corticectomy (1 case) and stereoelectroencephalography (SEEG) guided thermocoagulation. Fifty-one patients underwent a total of 378 SEEG electrodes implantation, and a median of 7 electrodes, ranging from 4~15 electrodes, were implanted per patient. The features of patients according to the PET-MRI imaging are summarized in Table 1.

**Table 1:** Patient features and characteristics of the PET-MRI co-registration imaging.

	BOSH	SIOS	SGOS	MGOS	*p*
Sex (M/F)	7/2	2/2	22/13	20/15	*p* = 0.703
Age at epilepsy onset	6.64 ± 4.20	6.93 ± 6.86	6.12 ± 4.76	10.95 ± 8.12	*p* = 0.068
Epilepsy duration	9.81 ± 10.09	7.31 ± 9.61	10.21 ± 6.77	9.29 ± 6.43	*p* = 0.695
Age at surgery	16.44 ± 10.57	14.23 ± 12.47	16.33 ± 8.40	20.24 ± 9.71	*p* = 0.285
**Seizure frequency**					
Daily	7	4	19	9	*P* = 0.006^*^
Weekly	2	0	11	11	
Monthly	0	0	3	14	
Yearly	0	0	2	1	
**Past history**					
Birth injury	0	0	2	2	*p* = 0.420
Febrile convulsion	0	0	5	4	
Encephalitis	1	0	3	1	
Trauma	0	0	1	4	
**Localization**					
Frontal lobe	9	4	20	6	*P* < 0.001^*^
Temporal lobe	0	0	2	18	
Parietal lobe	0	0	9	5	
Occipital lobe	0	0	1	5	
Insula	0	0	3	1	
**Pathology**					
FCD I	1		4	16	*P* = 0.001^*^
FCD IIa	2	4	15	12	
FCD IIb	6		16	7	

^*^Significantly different.

### MRI, PET and PET-MRI co-registration in FCD detection

**Figure 2:**
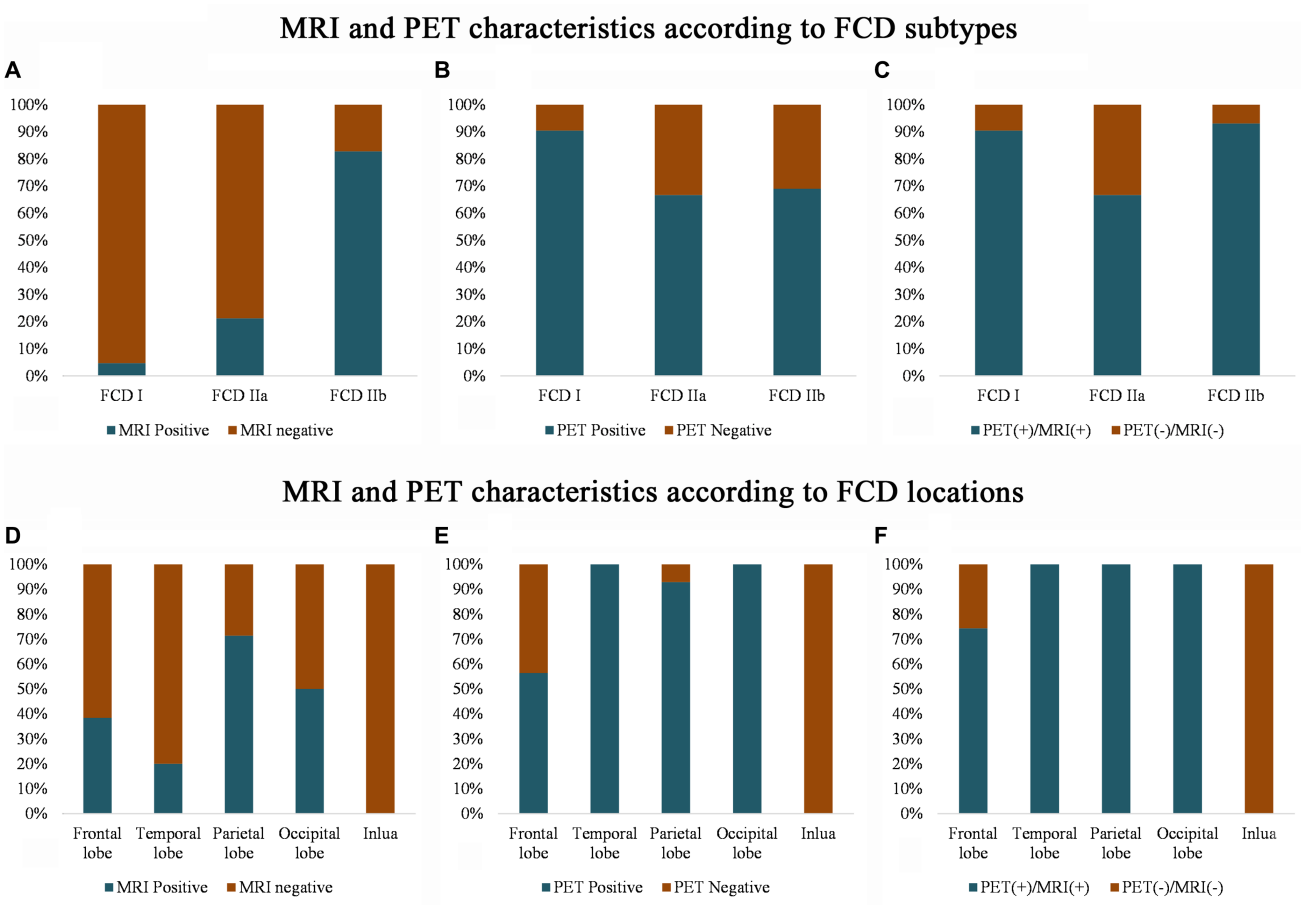
MRI and PET imaging characteristics according to the FCD histological classification **(A–C)** and cerebral location **(D–F)**. Note that compared with the MRI scans, PET has a higher sensitivity for detecting FCD type I **(B)** and temporal FCD **(E)**. Even with the MRI and PET scans, several frontal and insular FCDs were missing **(F)**, and most of the FCDs were FCD type IIa **(C)**. PET (+)/MRI (+) means that either the MRI or the PET imaging shows an abnormality, and PET (−) and MRI (−) means that neither the MRI nor the PET imaging detects any abnormality after the visual assessment.

The imaging features of the MRI and PET scans are presented in Figure 2. Conventional visual analysis showed PET scan (72%) was more sensitive in FCD detection than MRI (39%), especially in temporal lobe (100%) and FCD type I (90.5%); the detection rate of PET scans (66.7%) for FCD type IIa was also significantly higher than that of MRI (21.2%, *p* < 0.001) and no difference was found for FCD type IIb detection between MRI and PET (82.8% vs. 69.0%. *p* > 0.05). Both PET and MRI conventional visual analysis had limited power for insular FCD detection.

**Figure 3:**
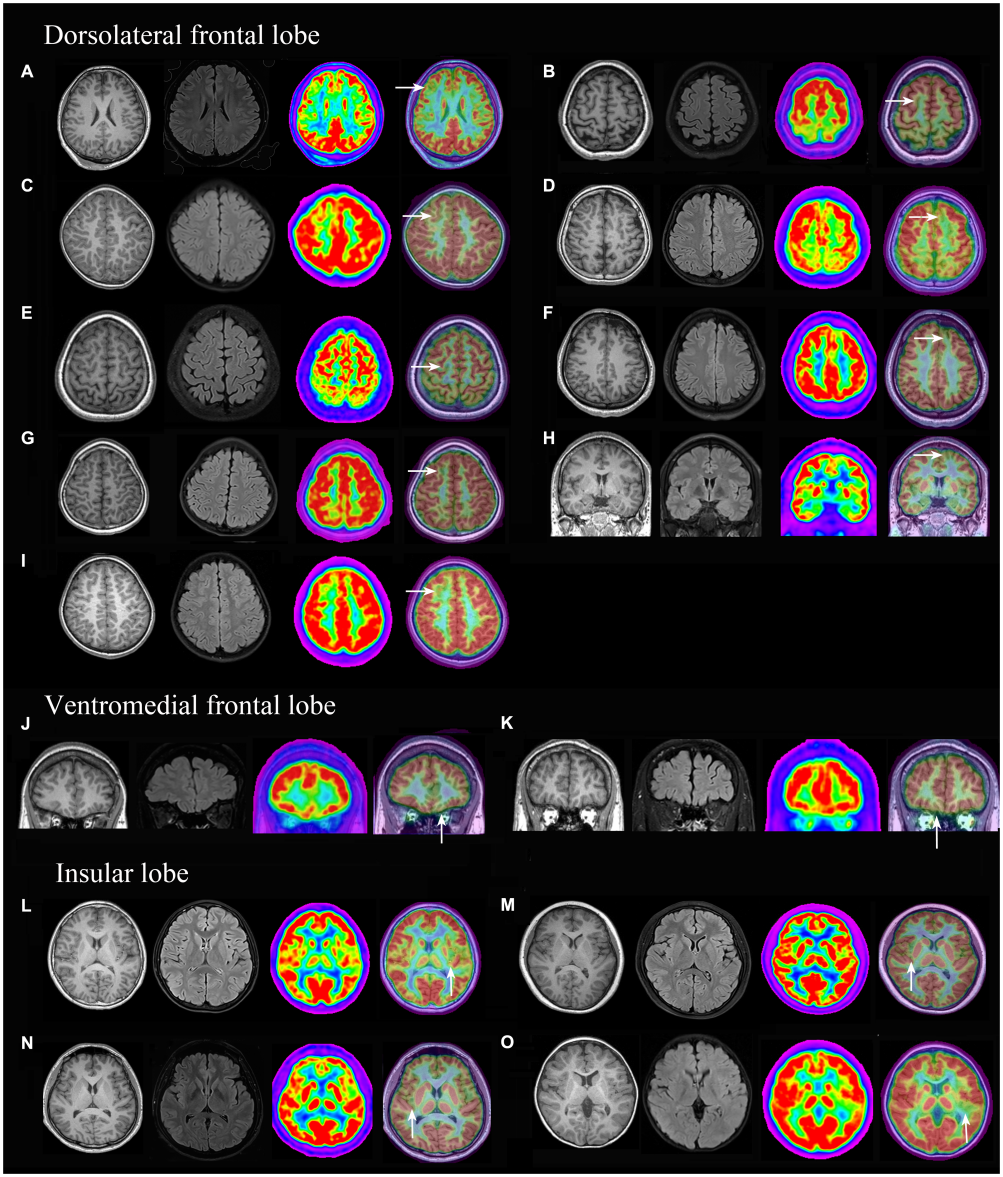
3D-T1, Flair and PET-MRI co-registration images of patients with PET (-) and MRI (-) FCD. According to the pathological diagnosis, images **(A,K)** were histologically confirmed as FCD type I, images **(D,I)** as FCD IIb and the other images **(B, C, E, F, G H, J, L, M, N and O)** were FCD IIa.

PET-MRI co-registration detected additional 15 cases (Figure 3) that were both MRI and PET negative and anatomically included the ventromedial frontal lobe (2 cases), dorsolateral frontal lobe (9 cases, 8 cases were located in a single sulcus,) and insular lobe (4 cases); histologically, the cases included FCD types I (2 cases), IIa (11 cases, Supplementary Figures e1, e2), IIb (2 cases).

### Clinical characteristics of PET-MRI co-registration hypometabolic patterns

Hypometabolic patterns with no MGOS (focalized pattern) were primarily observed in frontal lesions (33/39) and FCD types IIa (21/33) and IIb (22/29), and patterns with MGOS (extensive pattern) were primarily observed in the temporal lobe (18/20) and FCD type I (16/21). The differences in the hypometabolic patterns were significant between the frontal lobe and temporal lobe (*p* < 0.001) and between FCD types I and II (*p* = 0.001). These results indicated that frontal lobe FCD and FCD type II were more focalized than that in temporal lobe and FCD type I, respectively (Table 1).

**Figure 4:**
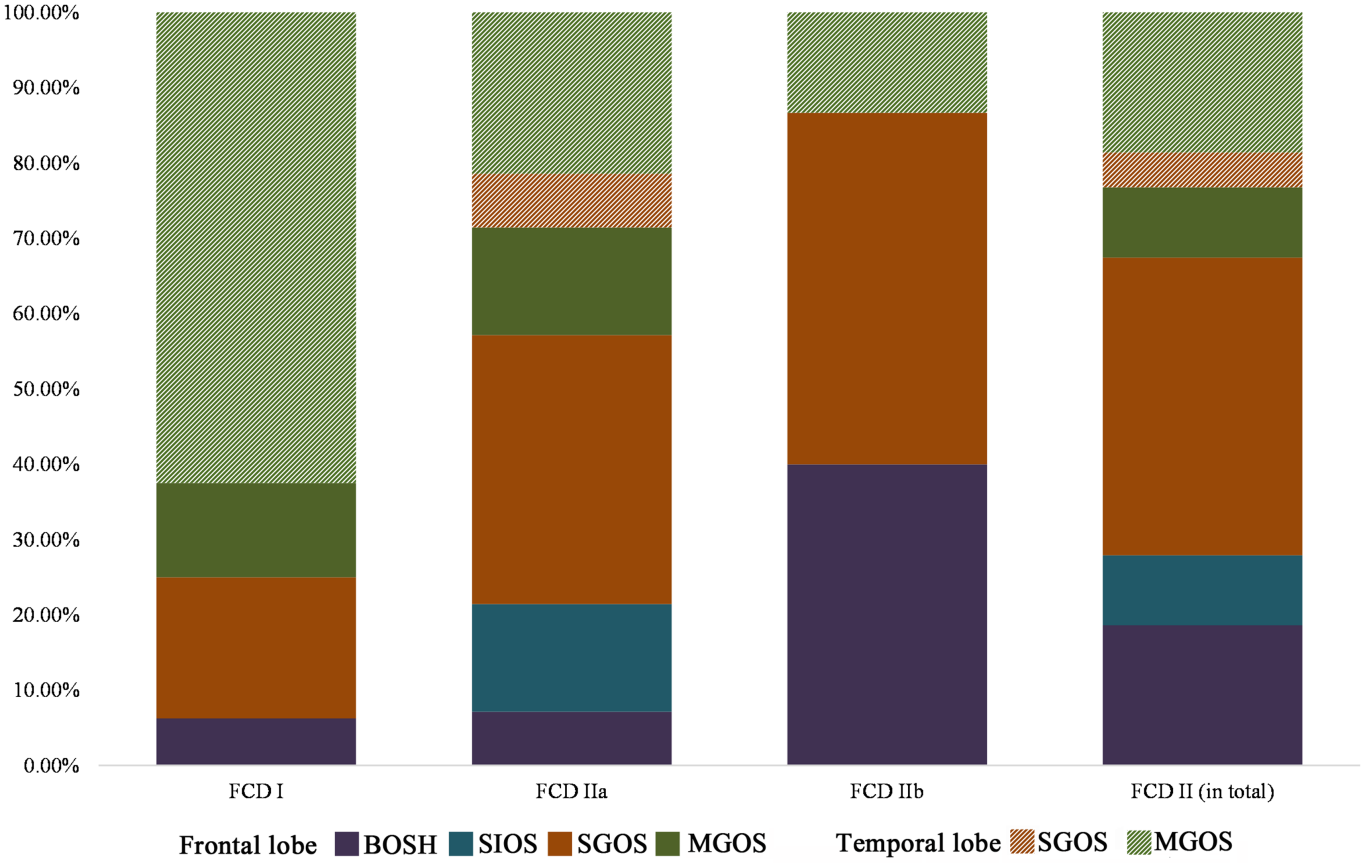
Lobar characteristics of frontal and temporal FCD in the PET-MRI co-registration imaging (FCD types I: *n* = 16, IIa: *n* = 28, IIb: *n* = 15). No BOSH and SIOS were found in temporal lobe FCD. Note that FCD type II was more prevalent in frontal lobe than that in temporal lobe and hypometabolic patterns of temporal lobe FCD were more extensive (MGOS) than those of frontal lobe FCD (BOSH, SIOS and SGOS).

All cases with FCD type I in the temporal lobe were MGOS (10/10), while non-MGOS types were prominent in the frontal lobe (4/6), and this difference was significant (*p* = 0.008). A significant difference was also found in terms of FCD type IIa in the frontal and temporal lobes (non-MGOS/total: 16/20 vs. 2/8, *p* = 0.011). Rare cases with FCD type IIb in temporal lobe limited the comparison of hypometabolic patterns between temporal and frontal lobe. These results indicated that for one specific FCD histological subtype, frontal lobe lesions tended to be more focalized than that in temporal lobe (Figure 4).

The preliminary univariate analysis showed that significant differences in terms of hypometabolic patterns were found for seizure frequency (*p* = 0.006), localization (*p* < 0.001) and FCD subtypes (*p* = 0.001). Then, the above-mentioned variables were included in the multiple logistic regression analysis (R^2^ = 0.613 [Cox & Snell], 0.686 [Nagelkerke], 0.425, [McFadden]; Model χ^2^(33) = 78.718; *p* < 0.001). We found an overall association between the hypometabolic patterns and the FCD location [χ^2^(12) = 36.898; *p* < 0.001] and histological subtype [χ^2^(6) = 14.267; *p* = 0.027]. There was no significant association with seizure frequency [χ^2^(9) = 9.084; *p* = 0.430].

### Surgical outcome

All the patients have more than 2 years’ follow-up, with 78.3% of the patients achieved seizure freedom. No significant association was found between different hypometabolic patterns and surgical outcome.

## Discussion

PET-MRI co-registration imaging offers a better delineation of the abnormal PET focus in relation to the underlying and neighboring anatomy and increases the detection rate of FCD. In this study, we found that incorporating PET-MRI co-registration into the pre-surgical evaluation of patients enhanced the sensitivity of detecting FCD type IIa. We also found that different FCD histological subtypes and cerebral location were significantly associated with different hypometabolic patterns.

### PET-MRI co-registration improves the detection of FCD type IIa

The sensitivity of the MRI detection of FCD is controversial because the diagnostic criteria of “MRI negative” are slightly different depending on the type of MRI technology and the experience of the interpreter (Salamon et al., 2008). The current study showed PET scan was more sensitive in FCD detection than MRI, which was consistent with previous reports (Park et al., 2006; Kim et al., 2009; Lerner et al., 2009; Chassoux et al., 2012; Halac et al., 2016). Highest positive rates of temporal lobe FCD (100%) and FCD type I (90.5%) were revealed in convention PET visual analysis and more prevalent MGOS in theses subgroups might account for this findings. Furthermore, the PET-MRI co-registration imaging detected an additional 15 cases of FCD that were both MRI and PET negative and mostly located in a single sulcus in the dorsolateral frontal lobe. Most of the cases were FCD type IIa (11/15). These focal hypometabolic patterns were easily ignored by convention visual analysis because of limited structural resolution in PET imaging.

### The association between hypometabolic patterns and the FCD classification

Here, we first classified the PET-MRI co-registration hypometabolic patterns into BOSH, SIOS, SGOS, and MGOS. Our results confirmed that compared to the focalized patterns of FCD type II, the hypometabolic patterns of FCD type I were more extensive, which were consistent with previous studies in which patients with FCD type I tended to have multilobar or unilobar resections, while more patients with FCD type II underwent a corticectomy (Krsek et al., 2009; Kwon et al., 2016).

Thus far, no mechanism could precisely explain these findings. A whole-brain MRI phenotyping analysis showed that a frontal lobe epilepsy with FCD type I displayed cortical thinning, but there was cortical thickening in FCD type II (Hong et al., 2016). The thickening patterns in FCD type II were hypothesized to result from delayed pruning at a late stage of cortical development, and the thinning patterns in FCD type I might relate to an abnormal tangential migration (Hong et al., 2016). The hypothesis that neurons that move parallel to the brain surface during tangential migration often transgress regional boundaries (Nadarajah and Parnavelas, 2002) may contribute to the extensive patterns in FCD type I. The MRI imaging evidence of FCD type II, including the transmantle sign (Barkovich et al., 1997), arrangement along an axis perpendicular to the cortex and a gradient of decreasing abnormal signal intensity from the bottom of the dysplastic sulcus to the surrounding gyri, was consistent with a disruption of the radial neural migration (Mellerio et al., 2012). This radial neural migration disorder may contribute to the focalized pattern in FCD type II.

### The association between hypometabolic patterns and the FCD location

Our study also found that frontal FCDs were more preferentially focalized than the temporal FCDs. Previous studies (Colombo et al., 2012; Mellerio et al., 2012) in which FCD type II was predominantly located in the frontal lobe may explain this phenomenon. However, the hypometabolic patterns in FCD types I and IIa were extensive in the temporal lobe but focalized in the frontal lobe. Different pathomechanisms of specific FCD histological subtypes may still exist between the frontal and temporal lobes. Haas et al. found architectural differences associated with FCD between the temporal and frontal lobes. They found that compared with the temporal group, additional accumulations of abnormal SMI32-positive pyramidal neurons were observed in layer 3 and were displaced in layer 4, indicating that frontal lobe FCD was more severely affected (Fauser et al., 2013). The difference in the mechanism underlying the development of frontal and temporal lobe FCD requires further research.

### FCD reading in PET-MRI co-registration: enlightenment from hypometabolic patterns’ classification

Among 35 patients with SGOS, 33 patients presented hypometabolic areas involving two islands and the bottom of single sulcus and only 2 patients were characterized with single gurus hypometabolism which included superficial cortex of a gyrus and proximal continuous islands of sulci. Superficial cortex hypometabolism presents incontinuity in conventional PET imaging, while simple sulcus hypometabolism can be easily missed if the sulcus is narrow or small. PET-MRI co-registration could provide gray matter background and improved the detection sensitivity of such kind of hypometabolic pattern. All patients with BOSH, SIOS and most of SGOS were confined to a single sulcus and most (46/48) were extra-temporal lesions, especially in frontal lobe (33/48). In the revision of the Barkovich classification (Barkovich et al., 2005), bottom-of-sulcus dysplasia (BOSD) was defined as focal cortical dysplasia with balloon cells, which corresponded to FCD type IIb. BOSD was reported to have a predilection for the frontal lobe (Harvey et al., 2015; Nakajima et al., 2016). Consistently with previous reports, all cases with BOSH in our study were frontal lobe FCD, and 6 of 9 patients were FCD type IIb. These findings confirmed the detection value of PET-MRI co-registration in frontal lobe FCD. The predilection of the frontal lobe for most limited hypometabolic patterns reminds us to pay attention to the small focalized FCD in patients suspicious of frontal lobe epilepsy.

### Study limitations

The first limitation of our study was the patient selection bias, because only patients with both MRI and PET scans were included in our study and some FCD patients with obvious MRI lesions might have no PET imaging data. Secondly, hypometabolic patterns of PET-MRI co-registration were evaluated subjectively. Actually, we could not confirm the extension of hypometabolic area was accord with the pathological lesion, especially in FCD type I. Thirdly, the few patients with FCD in the parietal, occipital and insular lobes and patients with hypometabolic patterns of BOSH and SIOS limited further statistical analysis; FCD type I was not further classified into Ia, Ib, and Ic as defined by ILAE (Blümcke et al., 2011). Fourthly, other presurgical evaluation information, including seizure semiology and EEG, were not analyzed together with imaging analysis, which are important predictors for surgical outcomes. Lastly, While FDG-PET is used in clinical practice for epilepsy surgery, PET with other specific ligands, including the AMPA (α-amino-3-hydroxy-5-methyl-4-isoxazole propionic acid) receptors (Sukprakun and Tepmongkol, 2022) and [(11)C] flumazenil (FMZ; Hodolic et al., 2016), may provide further Supplementary information for FCD localization.

## Conclusion

In conclusion, compared with the sensitivity of MRI in FCD detection, PET scans reinforce the detection of FCD type I, and PET-MRI co-registration further increases the detection of FCD type IIa, especially in frontal lobe. Focalized patterns, including BOSH, SIOS, SGOS, were mostly observed in FCD type II and frontal lobe lesions, while MGOS was more frequently presented in FCD type I and temporal lobe lesions. Furthermore, the predilection of focalized hypometabolic patterns in frontal lobe FCD suggested that subtle lesions should be checked carefully in patients suspicious of frontal lobe epilepsy.

## Data availability statement

The original contributions presented in the study are included in the article/Supplementary material, further inquiries can be directed to the corresponding author.

## Ethics statement

The studies involving humans were approved by Institutional Review Boards of Beijing Tian Tan Hospital. The studies were conducted in accordance with the local legislation and institutional requirements. Written informed consent for participation in this study was provided by the participants’ legal guardians/next of kin.

## Author contributions

XW and WH: acquisition of data, statistical analysis, and drafting the manuscript. XS and ZZ: acquisition and interpretation of data and revising the manuscript for intellectual content. LA, CZ, and LS: acquisition of data and revising the manuscript for intellectual content. J-gZ: acquisition and interpretation of data. KZ: study design, study supervision, and final revising the manuscript for intellectual content. All authors contributed to the article and approved the submitted version.
