# Supplementary material for: Hypometabolic patterns of focal cortical dysplasia in PET-MRI co-registration imaging: a retrospective evaluation in a series of 83 patients
Source: Front Neurosci. 2023 Sep 25;17:1173534. doi: 10.3389/fnins.2023.1173534 (PMC10561385; doi:10.3389/fnins.2023.1173534)
Supplement: Supplementary file 1 [file Data_Sheet_1.docx]

Supplementary Material

Hypometabolic patterns of focal cortical dysplasia in PET-MRI co-registration imaging: a retrospective evaluation in a series of 83 patients

**Xiu Wang^1,c^; Wenhan Hu^2,6,†^; Xiaoqiu Shao3; Zhong Zheng^4^; Lin Ai^5^; Lin Sang^4^; Chao Zhang^1,6^, Jian-guo Zhang^1,2,6^; Kai Zhang^1,6^.**

**Corresponding author:** Kai Zhang, Department of Neurosurgery, Beijing Tian Tan Hospital, Capital Medical University, Beijing, China, 100070,**E-mail:** [zhangkai62035@163.com](mailto:zhangkai62035@163.com)

**1. Cases series**

Figure e-1 and e-2: Case history, imaging and SEEG information in two typical patients with frontal lobe FCD, which were easy to be overlooked with conventional MRI and PET reading.


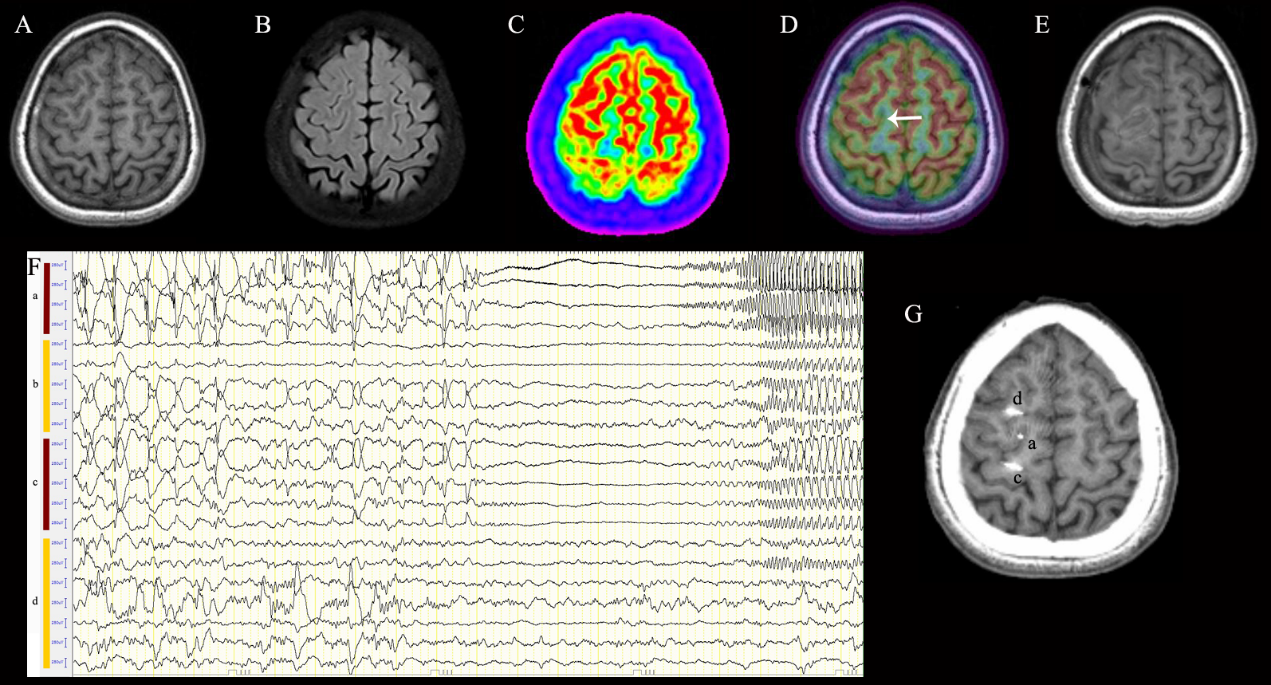


Figure-e1: FCD type IIa that was difficult to identify by the conventional MRI (A, B) and PET (C) visual analysis. This 12-year-old boy presented with seizures that began at the age of 8 years. The seizures were characterized by tonic events involving the left arm, followed by asymmetric tonic posturing (left elbow extension, right elbow flexion). The interictal scalp EEG disclosed a paroxysmal spike in the right frontal and central regions. The ictal events were associated with EEG changes in the right frontal, central and parietal lobes. The PET-MRI co-registration showed a small focalized hypometabolism in a sulcus in the right pre-motor area (D, white arrow). The ictal onset zone in the SEEG signals corresponds to the hypometabolic sulcus (electrode a) with a rapid low voltage discharge, followed by preictal spiking (F, G). Three of the 4 electrodes are shown in the co-registration image of the pre-surgical MRI and the post-SEEG implantation CT imaging (G), and electrode b, which was implanted from the right inferior precentral gyrus and targeted the right cingulated motor area, is not shown in figure G. The patient has been seizure-free for 14 months after a focal resection of the hypometabolic area (E).


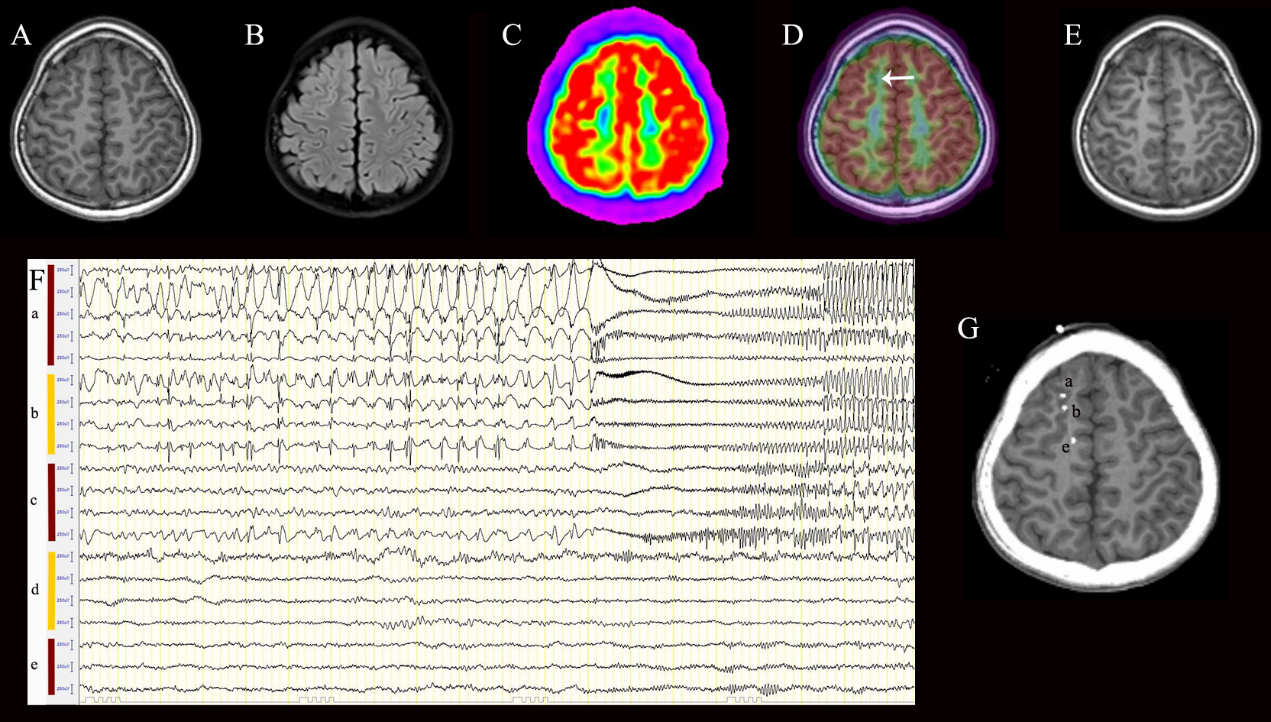


Figure-e2: Right frontal FCD type IIa with a negative MRI (A, B) and PET (C) by the conventional visual analysis. A 6-year-old child presented with seizures since the age of 3 years. The onset semiology was initially characterized by gelastic seizures and then changed into ictal fear, which was frequently present at midnight. The interictal scalp EEG disclosed spike waves in the bilateral frontal and central regions. The ictal events were associated with EEG changes in the midline and right lateral frontal lobe. The PET-MRI co-registration showed a small focalized hypometabolism in the middle part of the right superior frontal sulcus (D, white arrow). The ictal onset zone in the SEEG signals corresponded to the hypometabolic sulcus (electrode a) with preictal spiking with rhythmic spikes of low frequency, followed by a low-voltage fast activity (F, G). Three of the 5 electrodes are shown in the co-registration image of the pre-surgical MRI and post-SEEG implantation CT imaging (G), electrode c targeted the right parietal area (PET imaging showed hypometabolism in this area) and electrode d, which targeted the right orbitofrontal gyrus, is not shown in figure G. The patient was seizure free for 19 months after a focal resection of the hypometabolic area (E).
